# Latex-Based Polystyrene Nanocomposites with Non-Covalently Modified Carbon Nanotubes

**DOI:** 10.3390/polym13071168

**Published:** 2021-04-05

**Authors:** Jae Phil Song, Sung Ho Choi, Dae-Won Chung, Seong Jae Lee

**Affiliations:** Department of Polymer Engineering, The University of Suwon, Hwaseong, Gyeonggi 18323, Korea; sjphil@suwon.ac.kr (J.P.S.); shchoi@suwon.ac.kr (S.H.C.); dwchung@suwon.ac.kr (D.-W.C.)

**Keywords:** nanocomposites, carbon nanotube, noncovalent modification, rheological properties, electrical properties

## Abstract

We prepared electrically conductive polystyrene (PS) nanocomposites by incorporating non-covalently surface-modified carbon nanotubes (CNTs) with hydrophilic polymers such as polydopamine (PDA) and poly(3,4-ethylenedioxythiophene):poly(styrenesulfonate) (PEDOT:PSS). Further, ethylene glycol (EG) was introduced as a second dopant to improve the electrical properties of the nanocomposites prepared with PEDOT:PSS-wrapped CNTs. All conductive PS nanocomposites were prepared through latex-based process, and the morphology and properties of the nanocomposites were investigated. The electrical properties of the nanocomposites with PEDOT:PSS-wrapped CNTs were better than those of the nanocomposites with PDA-coated CNTs owing to the conducting nature of PEDOT:PSS, although the dispersions of both types of modified CNTs in the PS matrix were excellent, as evidenced by morphology and rheology. In the case of PEDOT:PSS modification, the electrical properties of the nanocomposites with EG-doped PEDOT:PSS-wrapped CNTs were superior to those of the nanocomposites without EG treatment.

## 1. Introduction

Polymer nanocomposites, i.e., nanofiller polymer composites, have attracted considerable attention recently because of their potential uses in high-performance materials and electronic devices [1]. This is mainly because the nanocomposites have superior mechanical and electrical properties to conventional composites. In particular, conductive polymer nanocomposites are very promising materials with excellent electrical properties of the conductive nanofiller and excellent characteristics of the polymer, such as the low cost, light weight, and ease of processing. Nanofillers may be classified according to the dimensionality of the nanostructure, i.e., zero-, one-, two-, or three-dimensional (0D, 1D, 2D, or 3D, respectively). Among them, 1D nanofillers, such as nanotubes, nanorods, and nanowires, have smaller dimensions and a higher aspect ratio than 2D and 3D nanofillers. Moreover, 1D nanofillers can efficiently transport electrical carriers along one controllable pathway, resulting in the conductivity enhancement of the nanocomposites [2,3,4].

Carbon nanotubes (CNTs) are among the most fascinating 1D nanofillers and have been known for their remarkable mechanical, electrical, and thermal properties since their discovery in 1991 [5]. When CNTs are used as nanofillers of polymer nanocomposites, they can significantly improve the properties of the matrix polymer with small addition, but aggregation occurs, which is one of the obstacles in dispersion, owing to the van der Waals interaction between CNTs. Thus, to improve the dispersion of CNTs in polymers, chemical and physical modifications of CNTs have been performed, such as hydrophilic and organophilic functionalization, stabilization with surfactants, and encapsulation with polymers and biomolecules [6,7,8,9,10]. Chemical (covalent) modifications significantly reduce the electrical conductivity by altering the intrinsic properties due to the change in hybridization from sp^2^ to sp^3^, whereas non-covalent modifications such as π−π interactions between CNTs and conjugated polymers preserve the sp^2^ hybrid orbitals. Among the various modification methods, physical encapsulation techniques have the advantage of dispersing CNTs without destroying the intrinsic properties of raw CNTs and without degrading the mechanical properties of the polymer matrix. In this study, two representative polymers were employed to efficiently coat or wrap CNTs in a non-covalent fashion: Polydopamine (PDA), which is a mussel-inspired biopolymer, and poly(3,4-ethylenedioxythiophene):poly(styrenesulfonate) (PEDOT:PSS), which is an electrically conductive polymer. First, CNTs were coated with dopamine and polymerized to PDA to give them a hydrophilic nature for improving their dispersion in an aqueous system. PDA can be formed on a wide selection of inorganic and organic materials via the spontaneous oxidative polymerization of dopamine in an alkaline solution at room temperature [11]. This reaction not only provides facile and mild reaction conditions but also can be applied to any type of material [12,13]. The PDA layer can also be used as a versatile platform for secondary reactions [14]. In the development of organic rechargeable batteries, the PDA-coated CNTs can be a promising hybrid electrodes with enhanced rate-performance and excellent cycling stability [15]. Moreover, the PDA-coated surface becomes hydrophilic; thus, amphiphobic CNTs existing in bundles or aggregates can be transformed into individual hydrophilic CNTs in aqueous solution. Second, CNTs were wrapped with a conductive polymer PEDOT:PSS, which is a macromolecular salt consisting of two ionic polymers. The hydrophobic PEDOT backbone includes aromatic thiophene rings that enable strong π−π stacking interactions with hybrid carbons of the CNT surface. The hydrophilic PSS with a long molecular chain acts as a surfactant and wraps around the surface of the CNTs, dispersing them [16]. In addition, the electrical conductivity of PEDOT:PSS can be enhanced by extra treatment either with organic solvents such as ethylene glycol (EG) [16,17,18,19,20,21], dimethyl sulfoxide [18,21,22,23], and dimethylformamide [18,22] and a cosolvent [24] or with organic compounds such as polyalcohols [25,26,27], anionic surfactants [28], ionic liquid [29], and organic acids [30,31]. Although the mechanism is not yet well-understood, it is considered that the enhancement of the electrical conductivity by the organic solvents is based on the conformational change of the PEDOT backbone from a coiled (benzoidal) structure to a linear (quinoidal) structure [16]. This mechanism suggests that the electrical properties of the final nanocomposites can be tuned by controlling the conductivity of PEDOT:PSS with a second doping process.

For preparing polymer nanocomposites incorporated with CNTs, there are a variety of methods, such as melt blending [32], solution mixing [33], in situ polymerization [34], and latex-based process [35,36,37]. Among them, the latex-based process is a versatile technology for dispersing nanofillers such as CNTs and metallic nanowires together with colloidal polymer particles in an aqueous medium [38,39]. The advantages of this technology are that it is easy, reproducible, and reliable; above all, it provides good dispersion owing to the interpenetration between the CNTs and polymer particles. Thus, even when a small amount of CNT is added to the matrix polymer, excellent electrical conductivity can be reliably obtained. Additionally, the latex-based process does not require the use of toxic and flammable solvents, making this process safe and eco-friendly [36]. In this study, we prepared electrically conductive polystyrene (PS) nanocomposites with non-covalently modified CNTs using either PDA or PEDOT:PSS, via latex-based process, which involves freeze-drying the aqueous mixture of colloidal polymer particles and nanofillers and subsequent molding. The prepared nanocomposites were investigated to evaluate their morphology and rheological and electrical properties and to compare the efficiency of the modified CNTs.

## 2. Experimental

### 2.1. Materials

Styrene monomer (Samchun Chemical, Seoul, Korea) was purified by vacuum distillation to remove inhibitors. Deionized water and ethanol were used as dispersion media, and potassium persulfate (KPS, Samchun Chemical) was used as a water-phase initiator. CNTs (NC7000, multi-walled CNTs, Nanocyl, Sambreville, Belgium) were used as electrically conductive nanofillers. Dopamine hydrochloride (Sigma-Aldrich, St. Louis, MO, USA), tris(hydroxymethyl) aminomethane (Tris, Acros Organics, Geel, Belgium), and hydrochloric acid (Samchun Chemical) were used to form a PDA coating layer on the CNT surface. 3,4-ethylenedioxythiophene (EDOT, Sigma-Aldrich), PSS (70,000 *g* mol^−1^, 30 wt% aqueous solution, Sigma-Aldrich), iron(III) sulfate hydrate (IS, Sigma-Aldrich), and sodium persulfate (SPS, Samchun Chemical) were used to synthesize PEDOT:PSS. EDOT was purified via distillation under a reduced pressure before use. As a secondary dopant, analytical-grade EG was purchased from Sigma-Aldrich. All the other solvents and reagents were used without further purification.

### 2.2. Synthesis of PS Microparticles

Monodisperse PS particles approximately 500 nm in diameter were synthesized via emulsifier-free emulsion polymerization. The overall procedure for PS microparticle synthesis was slightly modified from that described previously [40]. Briefly, 40 mL of styrene as a monomer and 0.3676 g of KPS as an initiator were added to a three-neck double-jacket reactor containing 360 mL of deionized water and 40 mL of ethanol, which was equipped with a mechanical stirrer, a reflux condenser, and a nitrogen inlet. Polymerization was conducted at 70 °C for 24 h with an agitation speed of 300 rpm. The particles were purified through centrifugation at 10,000 rpm for 10 min with ethanol and water. This step was repeated three times.

### 2.3. Preparation of PDA-CNTs

**Figure 1:**
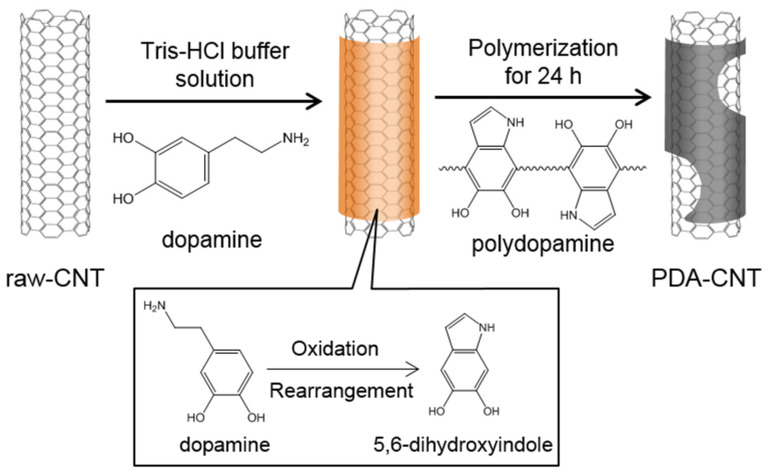
Schematic showing the procedure for the preparation of polydopamine-coated carbon nanotubes (PDA-CNTs).

CNTs (1.2 g) were added to 300 mL of a Tris-HCl buffer solution (10 mM, pH = 8.5), followed by ultrasonication at 20 W for 30 min in an ice-filled water bath using an ultrasonic probe (VC505, Sonics, Newtown, CT, USA). Here, the pH of the Tris-HCl buffer solution was measured using a potentiometric titrator (848 Titrino Plus, Metrohm, Herisau, Switzerland). Next, 1 g L^−1^ dopamine hydrochloride was added to the CNT suspension. The CNT and dopamine mixture was stirred at room temperature for 24 h. The color of the mixture changed to dark brown because of the spontaneous oxidative polymerization of dopamine. To remove the non-reactants, the PDA-coated CNTs were filtrated, thoroughly washed with deionized water in a glass filter funnel three times, and dried in a vacuum oven at 50 °C for 24 h. Finally, the CNTs partially coated with a thin PDA layer (PDA-CNTs) were obtained and used for the subsequent experiments. Figure 1 schematically shows the procedure for PDA-CNT preparation.

### 2.4. Preparation of PEDOT:PSS-CNTs

**Figure 2:**
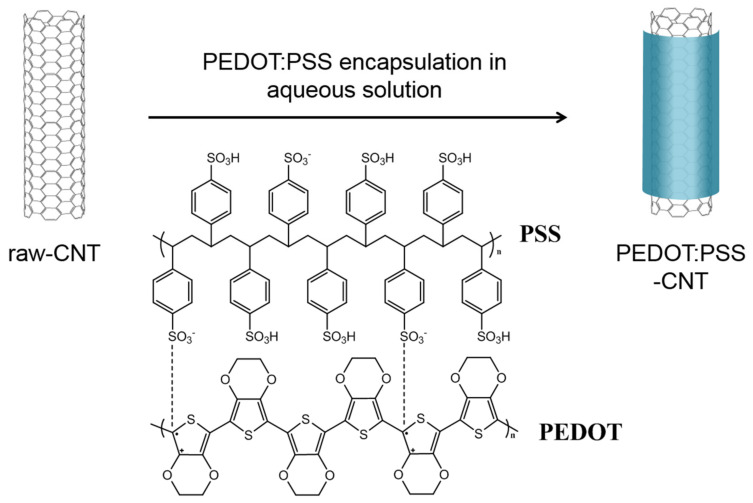
Schematic showing the procedure for the preparation of poly(3,4-ethylenedioxythiophene):poly(styrenesulfonate)-wrapped carbon nanotubes (PEDOT:PSS-CNTs).

PEDOT:PSS was synthesized to compare the electrical conductivity and hydrophilicity effects of PEDOT:PSS by adjusting the ratio of PEDOT to PSS for future studies. The preparation of PEDOT:PSS-CNTs consists of two steps: The synthesis of PEDOT:PSS and the encapsulation of PEDOT:PSS around the CNT surface [16]. First, the conducting polymer PEDOT:PSS was synthesized via the oxidative polymerization of EDOT. EDOT (3.52 g, 24.8 mmol) and a 30 wt% PSS aqueous solution (30.4 g, 49.6 mmol) were mixed with 150 mL of distilled water at a molar ratio of 1:2. Apart from this, SPS (5.90 g, 24.8 mmol) and IS (0.05 g, 0.125 mmol) were added to 150 mL of distilled water to prepare an oxidant solution. PEDOT:PSS was synthesized via dropwise addition of the oxidant solution to the EDOT:PSS aqueous solution at 25 °C for 24 h in a double-jacket reactor. The reaction mixture was treated twice with ion exchange resins and the content of PEDOT:PSS in aqueous solution was adjusted to 1.5 wt% by vacuum evaporation of water. Then, 0.5 g of CNTs was added to 40 mL of a 1.5 wt% PEDOT:PSS aqueous solution. The mixture was ultrasonicated at 20 W for 30 min using an ultrasonic probe and again for 1 h using an ultrasonic bath (KY 053, Kyung Il Ultrasonic, Ansan, Korea). Then, the aqueous solution containing PEDOT:PSS-wrapped CNTs (PEDOT:PSS-CNTs) was filtered and washed with deionized water. Subsequently, the rinsed PEDOT:PSS-CNTs were dried in a vacuum oven at 50 °C for 24 h and then at room temperature for 48 h. Figure 2 schematically shows the procedure for preparing the PEDOT:PSS-CNTs. EG-doped PEDOT:PSS-CNTs (EG-PEDOT:PSS-CNTs) were prepared in a similar way. Before the addition of the CNTs to the PEDOT:PSS aqueous solution, EG (5 wt%) was added to a 1.5 wt% PEDOT:PSS solution, followed by stirring for 2 h. The subsequent procedures were the same as those described above. To evaluate the electrical properties of the PEDOT:PSS and EG-PEDOT:PSS films, spin-coating tests were conducted using a spin coater (Ace-200, Dong Ah Trade Corp, Seoul, Korea). The solution used in the spin-coating test was prepared from a 1.5 wt% PEDOT:PSS (or EG-PEDOT:PSS) aqueous solution mixed with the same mass of isopropyl alcohol as the aqueous solution. This solution was spin-coated on an O_2_ plasma-treated slide glass at the coating speed of 600 and 2000 rpm for 45 s and dried in a convection oven at 120 °C for 1 min.

### 2.5. Preparation of PS/Polymer-Modified CNT Nanocomposites

**Figure 3:**
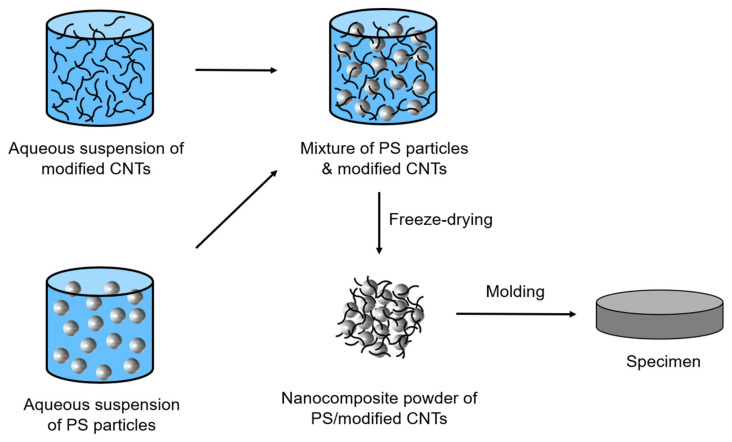
Schematic showing the procedure for the preparation of the polystyrene (PS)/polymer-modified CNT nanocomposite sample using latex-based process.

PS nanocomposites incorporated with non-covalently polymer-modified CNTs such as PDA-CNTs, PEDOT:PSS-CNTs, and EG-PEDOT:PSS-CNTs, were prepared via latex-based process. First, polymer-modified CNTs were dispersed in deionized water via ultrasonication at 20 W for 30 min. Next, PS microparticles were added to the polymer-modified CNT suspension and again dispersed via ultrasonic treatment at 20 W for 30 min. Then, the PS/polymer-modified CNT suspension was freeze-dried using liquid nitrogen. The freeze-dried PS/polymer-modified CNT nanocomposite powder mixture was used to prepare a disk-shaped specimen 1 mm thick and 25 mm in diameter via hot pressing at 180 °C for 5 min. Figure 3 schematically shows the procedure for preparing the PS and polymer-modified CNT nanocomposite sample using a latex-based process.

### 2.6. Characterization

The materials, nanofillers, and nanocomposites were characterized via thermogravimetric analysis (TGA), Fourier transform infrared (FT-IR) spectroscopy, X-ray photoelectron spectroscopy (XPS), and field-emission scanning electron microscopy (FE-SEM). The morphology of the PS particles and the polymer-modified CNTs, as well as the fracture surface of the nanocomposites, was confirmed using FE-SEM (JSM 6700F, Jeol, Tokyo, Japan). The chemical components and structures of the polymer-modified CNTs were characterized via FT-IR spectroscopy (Spectrum Two FT-IR, PerkinElmer, Waltham, MA, USA) and XPS (K-Alpha Plus, Thermo Fisher Scientific, Waltham, MA, USA). The thermal stability of the polymer-modified CNTs was evaluated via TGA (STA 409, Netzsch, Selb, Germany) with a heating rate of 10 °C min^−1^ in N_2_ atmosphere. The rheological properties of the nanocomposites were analyzed at 210 °C using a rotational rheometer (MCR 300, Anton Paar, Graz, Austria). To confirm the linear viscoelastic range, the strain sweep test was performed first, and then the frequency sweep test was performed at a strain of 3%, which is in the linear viscoelastic range. The frequency sweep test yields the storage modulus, loss modulus, and complex viscosity. The electrical resistances of the PEDOT:PSS and EG-PEDOT:PSS films were measured using a four-point probe system (M4P-302, MS Tech, Hwaseong, Korea) with a source measurement unit (Keithley 2400, Keithley, Solon, OH, USA). The resistances were converted into sheet resistances, taking into account the correction factor [41]. The electrical resistance of the bulk nanocomposites was measured using a picoammeter (Keithley 6487, Keithley) and a digital multimeter (Fluke 189, Fluke, Everett, WA, USA). Prior to the measurement, silver paste (Elcoat P-100, CANS, Tokyo, Japan) was applied to attach the electrodes to both sides of the nanocomposite sample. From the measured resistance, the electrical conductivity was obtained using the following equation:

(1) $$\sigma =\frac{1}{\rho }=\frac{d}{RS}$$

where σ is the electrical conductivity, ρ is the electrical resistivity, R is the electrical resistance, d is the sample thickness, and S is the sample area.

## 3. Results and Discussion

### 3.1. Characterization of Materials

**Figure 4:**
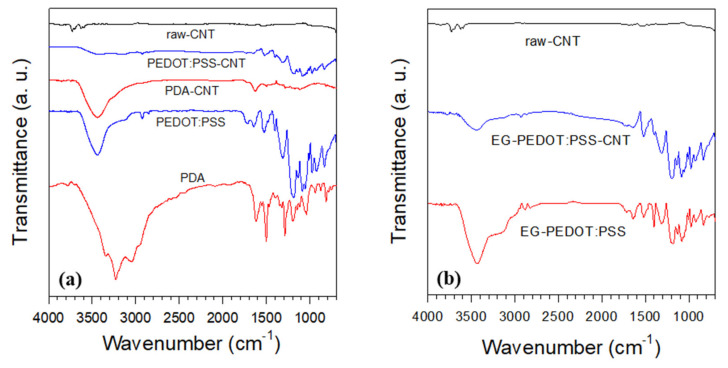
FT-IR curves of (**a**) raw-CNT, PDA, PEDOT:PSS, PDA-CNT, and PEDOT:PSS-CNT, and (**b**) ethylene glycol (EG)-PEDOT:PSS and EG-PEDOT:PSS-CNT.

The effects of the PDA coating and PEDOT:PSS wrapping on the CNT surface were observed via FT-IR spectroscopy. Figure 4a shows the FT-IR spectra of raw-CNT, PDA-CNT, PEDOT:PSS-CNT, PDA, and PEDOT:PSS. Comparing the spectra of raw-CNT, PDA-CNT, and PDA reveals that PDA-CNT exhibits the characteristic peaks of PDA (3420 cm^−1^ for the catechol OH bond and 1624 cm^−1^ for the aromatic C=C bond) [14]. The PEDOT:PSS wrapping around CNT is also confirmed from the spectra of raw-CNT, PEDOT:PSS-CNT, and PEDOT:PSS. The peaks at 1521 and 1308 cm^−1^; 975 and 833 cm^−1^; and 1182, 1133, and 1086 cm^−1^ are assigned to the C-C/C=C, C-S, and C-O-C from the PEDOT backbone, respectively [42,43]. Another peak at 1054 cm^−1^ is attributed to the SO_3_^-^ from the PSS [43]. Thus, it is concluded that the synthesis of PEDOT:PSS and the surface modification of CNTs by PEDOT:PSS were successfully performed. The FT-IR spectra of EG-PEDOT:PSS and EG-PEDOT:PSS-CNT are shown in Figure 4b. The EG-PEDOT:PSS exhibits similar peaks as those of PEDOT:PSS.

**Figure 5:**
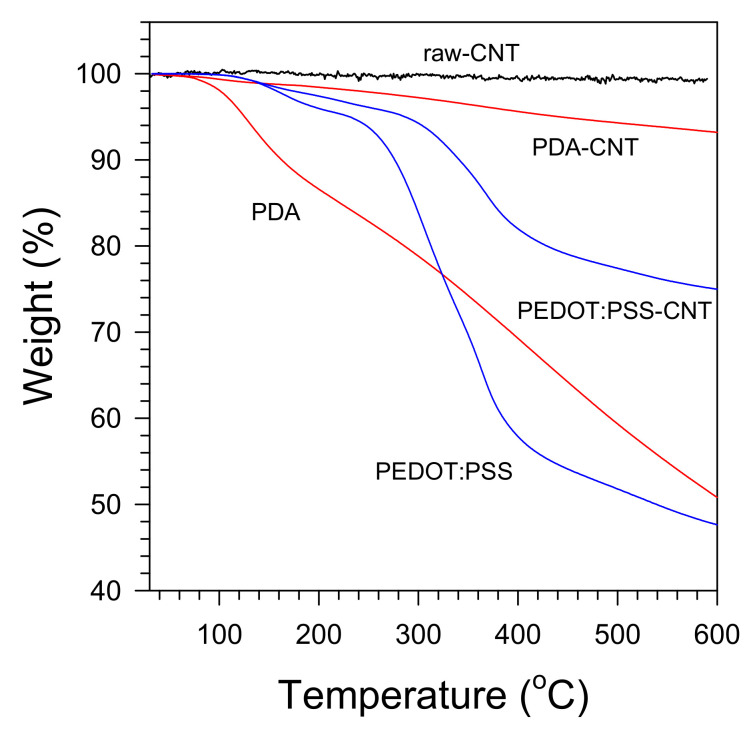
TGA curves of raw-CNT, PDA, PEDOT:PSS, PDA-CNT, and PEDOT:PSS-CNT.

Figure 5 shows the TGA results for quantitatively evaluating the degree of the PDA coating and PEDOT:PSS wrapping on the CNT surface. The raw-CNT did not exhibit detectable weight loss up to 600 °C. On the other hand, the PDA-CNT, which was surface-modified by the spontaneous oxidative polymerization of dopamine, was gradually reduced, showing a weight loss of 7% at 600 °C [12,44]. The TGA data for PDA indicate that the weight loss of PDA at 600 °C was ~49% of initial weight. From these results, we estimated that the weight of the PDA coated on the CNT was ca. 14.3% of the weight of the PDA-CNT [45]. Referring to the manufacturer’s technical data sheet, the average length and diameter of raw-CNTs are 1.5 μm and 9.5 nm, respectively. The density of the PDA is unknown but may be approximated with a density of 1.26 g cm^−3^ of dopamine. The density of CNTs varies with the diameter and number of walls of the CNTs. In the literature that estimated the density of CNTs [46], the density of CNTs used in this study can be estimated to be 1.3 g cm^−3^ based on the data (9.5 nm diameter, ~5 walls). By roughly neglecting the density difference between raw-CNTs and PDA, therefore we can estimate the average diameter of the PDA-CNTs as approximately 10.2 nm and the average thickness of the PDA layer as <0.4 nm. This is thinner than a single PDA layer, indicating that the raw-CNTs were not fully coated by PDA, i.e., it can be inferred that the PDA-CNT consists of conducting and insulating parts as schematically shown in Figure 1. This inference needs to be further confirmed by performing high-resolution TEM analysis. Consequently, the PDA-CNT had characteristics of both electrical conductivity (uncoated part) and hydrophilicity (coated part). The electrical conductivity of PS/PDA-CNT nanocomposites will be discussed in Section 3.4. The TGA curve of PEDOT:PSS is comprised of three sections: The first section of weight loss up to 220 °C, the second section between 220 and 410 °C, and the third section above 410 °C. The first loss section is ascribed to the loss of water adsorbed on the PSS. The second and third sections are ascribed to the decomposition of PSS through the rupture of the sulfonate group dissociated from styrene, and the decomposition of the polymer backbone, respectively [17,47,48,49]. The residual amounts of PEDOT:PSS and PEDOT:PSS-CNT at 600 °C were ~48% and ~75% of the initial weight, respectively. Thus, we estimated the amount of PEDOT:PSS in the PEDOT:PSS-CNTs to be approximately 48.1 wt%. A rough calculation indicates that the average layer thickness of PEDOT:PSS in PEDOT:PSS-CNT is slightly thicker than 2 nm.

**Figure 6:**
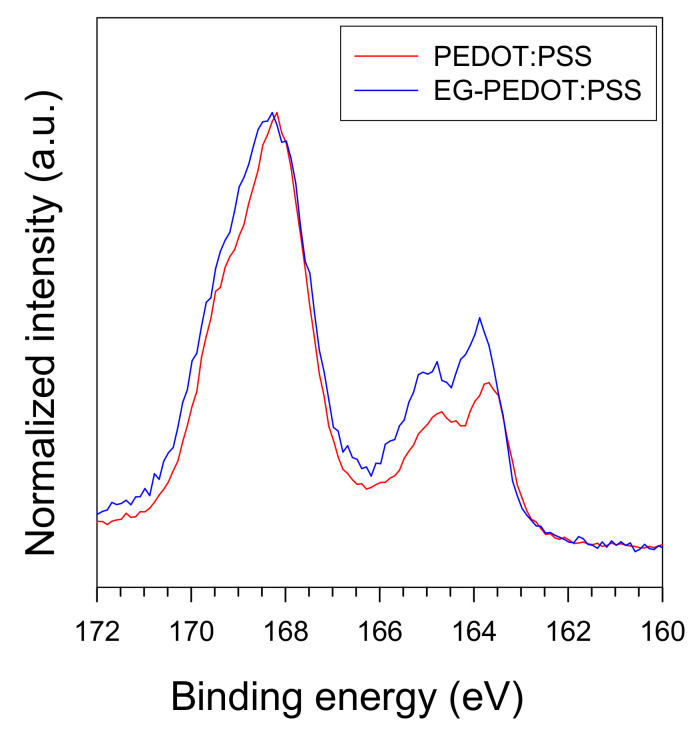
XPS spectra of PEDOT:PSS and EG-PEDOT:PSS.

The XPS spectra of sulfur 2p (S 2p) for pristine PEDOT:PSS and EG-treated PEDOT:PSS samples are shown in Figure 6. The lower-binding energy peaks at 163.5 eV correspond to the sulfur atom of the PEDOT. The higher-binding energy peaks near 168 eV correspond to neutral and ionic sulfur in the PSS dopant [22]. According to the height ratio of PSS to PEDOT for the pristine sample, the concentration of PSS dopants in PEDOT is estimated to be ~2.6. The high PSS dopant ratio of the samples originates from the increase of the number of dopants on the sample due to the relatively large size of the PSS chain. The ratio of PSS to PEDOT decreased to ~2.2 after the EG treatment, owing to the depletion of the PSS chains from the PEDOT:PSS [50]. The EG induced a screening effect between the positively charged PEDOT chains and negatively charged PSS chains, reducing the Coulomb interaction between them [22]. In addition to XPS, Raman spectroscopy is also very useful in chemistry to study the doping behavior of conjugated polymers [51,52]. The conformational change of the PEDOT chain in the PEDOT:PSS film caused by the EG treatment can be detected via Raman spectroscopy. As reported in the literature, the benzoid structure may be a preferred structure for the coil conformation of PEDOT:PSS, and the quinoid structure may be the preferred structure for the linear or expanded-coil conformation of EG-PEDOT:PSS [16,53]. Therefore, it can be inferred from XPS and Raman spectroscopy that higher PEDOT domains caused by the segregation of the excess PSS and the conformational change of the PEDOT chains from a coil structure to an extended coil or linear structure lead to higher conductivity. The sheet resistances of spin-coated PEDOT:PSS and EG-PEDOT:PSS films were measured, as summarized in Table 1. By adding a small amount of EG as an additional dopant, the sheet resistance of the PEDOT:PSS film was reduced to approximately1/20–1/50. The decreasing tendency of the sheet resistance was more pronounced when a higher EG content was used, and a lower coating speed was applied.

### 3.2. Morphology of PS/CNT Nanocomposites

**Figure 7:**
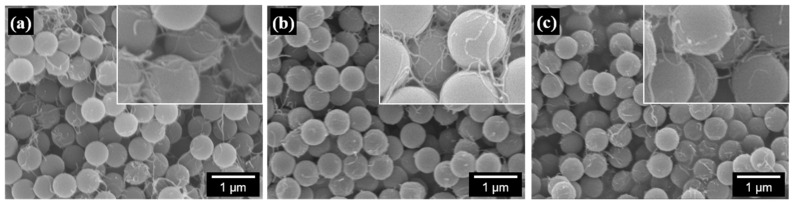
FE-SEM images of freeze-dried PS/CNT nanocomposite powder materials: (**a**) PS/PDA-CNT 3%, (**b**) PS/PEDOT:PSS-CNT 3%, and (**c**) PS/EG-PEDOT:PSS-CNT 3%. The scale bar represents 1 μm. The insets show 2.5 times higher magnification images.

**Figure 8:**
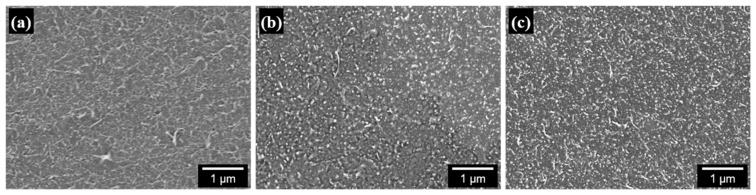
FE-SEM images of the fracture surface of compression-molded PS/CNT nanocomposite specimens: (**a**) PS/PDA-CNT 3%, (**b**) PS/PEDOT:PSS-CNT 3%, and (**c**) PS/EG-PEDOT:PSS-CNT 3%. The scale bar represents 1 μm.

The properties of PS/CNT nanocomposites are affected by many factors, such as the type and aspect ratio of CNTs, the molecular weight of PS chains, the size and uniformity of PS particles, and the degree of dispersion between PS particles and CNTs. In this study, the type and aspect ratio of CNTs are not controllable variables because we used only one type of commercial CNT. The average molecular weight and molecular weight distribution of the PS chains and the size and uniformity of the PS particles can also be considered fixed, as only one emulsifier-free emulsion polymerization technique with a specified recipe was employed. Therefore, the properties of the PS/CNT nanocomposites can be determined by the amount and degree of dispersion of CNTs in the PS matrix. Figure 7 shows SEM images of freeze-dried PS/CNT nanocomposite powder materials consisting of 500 nm PS particles and modified CNTs dispersed in an aqueous suspension. The PS particles can be regarded as having a monodisperse size, and the CNTs in all powder materials appear to be well-dispersed, either adsorbed on the PS particle surface or connected to each other. Figure 8 shows SEM images of the fracture surface of nanocomposite specimens that were compression-molded at 180 °C for 5 min using a hot press. Three SEM fracture images confirmed that CNTs were uniformly dispersed throughout the PS matrix. This excellent dispersion of CNTs is due to the latex-based process, which consists of mixing two components in an aqueous suspension and incorporates individual nanofillers into a highly viscous polymer matrix after freeze-drying and molding. The SEM images of the freeze-dried powder material and the fracture surface of the nanocomposite specimen for PS/EG-PEDOT:PSS-CNT were almost the same as those for PS/PEDOT:PSS-CNT, and the differences in the degree of dispersion were indistinguishable.

### 3.3. Rheological Properties of PS/CNT Nanocomposites

**Figure 9:**
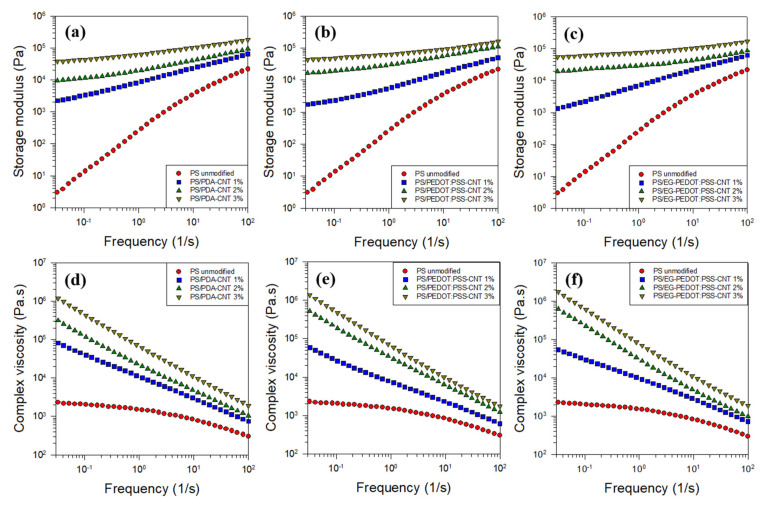
Rheological properties (storage modulus G′ and complex viscosity η*) of PS/CNT nanocomposites with (**a**,**d**) PDA-coated CNTs, (**b**,**e**) PEDOT:PSS-wrapped CNTs, and (**c**,**f**) EG-PEDOT:PSS-wrapped CNTs.

Since the rheology of the particulate dispersion system is influenced by the degree of dispersion of the added particles, the rheological properties of the PS/CNT nanocomposites can be used as a measure of dispersion. Figure 9 shows the rheological properties (storage modulus and complex viscosity) of PS/CNT nanocomposites incorporated with PDA-coated CNTs, PEDOT:PSS-wrapped CNTs, and EG-PEDOT:PSS-wrapped CNTs with respect to the angular frequency. The storage modulus of the nanocomposites increased with the frequency and CNT content because the materials under shearing action showed increasing solid-like behavior with the increase of the frequency and content. For unmodified PS (CNT-free), the complex viscosity shows a typical polymer melt rheology with Newtonian behavior, i.e., a constant viscosity at low frequencies and shear thinning behavior at higher frequencies. As the CNT content in three systems increased, the slope of the storage modulus became gentle, and that of the complex viscosity became steep. In linear polymer chains, it is well-known that the storage modulus G′ and loss modulus G″ at low frequencies ω show the relationship of G′~ω^2^ and G″~ω^1^. This behavior at low frequencies changes to G′~ω^1^ and G″~ω^0^ as the filler content increases owing to the suppression of the polymer chain relaxation by added fillers, resulting in the transition of materials from liquid-like to solid-like behavior [54]. Our results also followed this trend. The degree of dispersion of all modified CNTs in the PS matrix appears to be excellent. The addition of 1 wt% CNTs increased the storage modulus by a factor of approximately 700 at a low frequency of 0.03 s^−1^. Further addition of CNTs did not significantly affect the rheological properties compared with the initial addition of 1 wt% to the PS matrix. On the other hand, the increase in G′ and η* due to the addition of CNTs in the high frequency region is smaller than that in the low frequency region because the physical network between CNTs due to the higher shear force was destroyed and the rheological properties of the polymer matrix became important. Therefore, the difference in rheological properties between PS/CNT nanocomposites and the PS homopolymer was reduced in the high-frequency region. It is also noted that the G′ and η* graphs with respect to ω for the PS/EG-PEDOT:PSS-CNT nanocomposites were almost the same as those for PS/PEDOT:PSS-CNT counterparts.

**Figure 10:**
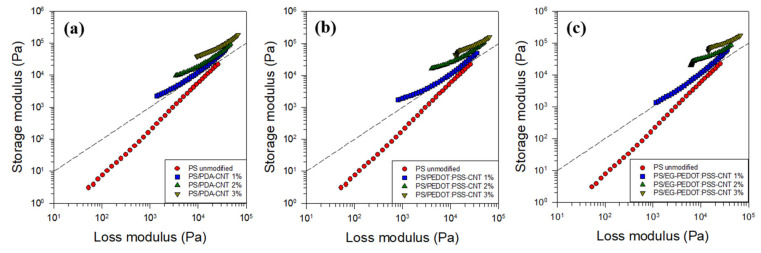
Storage modulus G′ vs. loss modulus G″ of PS/CNT nanocomposites with (**a**) PDA-coated CNTs, (**b**) PEDOT:PSS-wrapped CNTs, and (**c**) EG-PEDOT:PSS-wrapped CNTs. The dashed line represents G′ = G″ and is for visual guidance.

The log G′ vs. log G″ graphs of the PS/CNT nanocomposites are shown in Figure 10. This type of graph is called a modified Cole–Cole plot and is used to identify the structural differences in composites depending on the filler content [55]. Therefore, it is possible to predict the microstructural change of the PS/CNT nanocomposites by the shift of the graph and the change of the slope with respect to the CNT content. All three plots in Figure 10 show that the microstructure of the nanocomposites changed greatly when CNTs were added at a low concentration and at low frequencies. In addition, it can be inferred that a rheological network structure was formed, as the addition of 1 wt% CNTs caused G′ to be larger than G″ at all frequencies and the slope changed substantially in the graph, which is ascribed to the increase of the solid-like properties. Conclusively, the rheological percolation threshold in the PS/CNT nanocomposites considered in this study was close to or less than 1 wt%.

### 3.4. Electrical Properties of PS/CNT Nanocomposites

**Figure 11:**
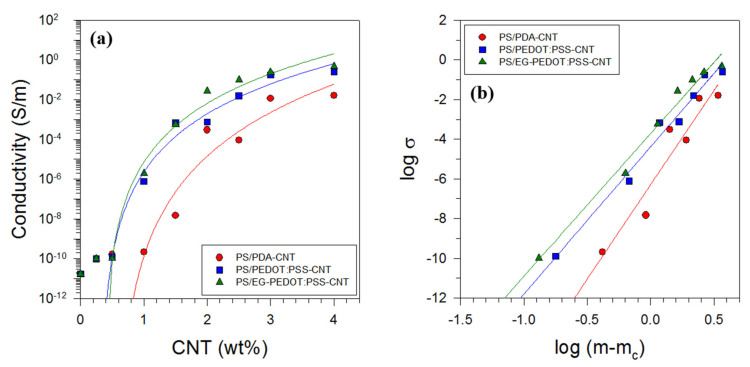
Effect of non-covalently polymer-modified CNTs on the electrical conductivity of PS/CNT nanocomposites: (**a**) electrical conductivity as a function of nanofiller content (The solid line represents the curve fitting by regression analysis of the power–law relationship given by Equation (2).) and (**b**) log–log plot of σ and *m*−*m_c_* (Each graph shows how nicely the regression fits the experimental data.).

The electrical conductivity of the PDA-CNT, PEDOT:PSS-CNT, and EG-PEDOT:PSS-CNT nanocomposites was measured to investigate the electrical properties of the nanocomposites. Figure 11 shows the electrical conductivity with respect to the modified CNT content for each nanocomposite. When small amounts of the conductive CNTs were added to the insulating PS matrix, the electrical conductivity did not change significantly (approximately 10^−11^–10^−10^ S m^−1^). After a certain content of CNTs was reached, the electrical conductivity increased rapidly, and as the content was continuously increased, the rate of increase decreased gradually. The point at which the electrical conductivity changes abruptly is the electrical percolation threshold, where the microstructure of the material begins to form a CNT network, i.e., an electrically conducting channel. At concentrations lower than the percolation threshold, the network structure is not formed, and electrons cannot flow, but at concentrations higher than the threshold, network structures can form, and electrons can flow. The electrical percolation threshold can be estimated using the following power–law relationship [54].

(2) $$\sigma \propto {\left(m-m_{c}\right)}^{b}$$

Here, m is the weight fraction of the modified CNTs, $m_{c}$ is the weight fraction at the threshold, and b is the critical exponent. Using Equation (2), the electrical percolation thresholds of the PDA-CNT, PEDOT:PSS-CNT, and EG-PEDOT:PSS-CNT nanocomposites were determined as 0.58, 0.32, and 0.36 wt%, respectively. The critical exponent b is related to the microstructural dimension of composites [56]. The b values higher than 2 have been observed for 1D fiber-filled systems [38,57,58]. The critical exponents of all nanocomposites prepared by latex-based process in this study were significantly higher than this value. Among the three nanocomposites, the dependence of the electrical conductivity on the CNT content was the lowest for the PDA-CNT sample and the highest for the EG-PEDOT:PSS sample. If CNTs were completely coated with PDA, the electrical conductivity of the nanocomposite would not improve much, but the conductivity was higher than expected as shown in Figure 11. It is believed that some electrical connections between the uncoated part of the CNTs have been formed. In the case of the PEDOT:PSS-CNT sample, the CNTs were wrapped with conductive PEDOT:PSS, indicating a higher electrical conductivity. Furthermore, PEDOT:PSS doped with EG had higher electrical conductivity than PEDOT:PSS, and the EG-PEDOT:PSS-CNT sample showed the largest electrical conductivity improvement. When the EG-PEDOT:PSS-CNT content reached 4 wt%, the electrical conductivity of the nanocomposite reached approximately 10^0^ S m^−^^1^. However, the effect of adding EG-PEDOT:PSS-CNT, which displayed better electrical conductivity than PEDOT:PSS-CNT, was not larger than expected. It is inferred that there is a potential for EG to evaporate at high temperatures during hot pressing for 5 min, although the molding temperature of 180 °C is lower than the boiling point of EG, 197.3 °C.

## 4. Conclusions

In order to develop a polymer/CNT nanocomposite with electrical conductivity, it is necessary to efficiently and effectively disperse the CNT bundles and agglomerates caused by van der Waals attraction while maintaining the electrical properties of the CNTs. The commonly used chemical modification is excellent to disperse CNTs but deteriorates their electrical properties, while non-covalent modification has the advantage of improving the dispersibility without degrading the intrinsic properties of the CNTs. In this study, we successfully prepared PS/CNT nanocomposites with enhanced electrical conductivity by uniformly dispersing the modified CNTs and PS particles via latex-based process. As a tool for evaluating the dispersibility and performance of nanofillers within a matrix, we compared the rheological properties of nanocomposites and also compared their electrical conductivity. CNTs were non-covalently modified into PDA-CNT through dopamine adsorption and polymerization, PEDOT:PSS-CNT wrapped with a conductive polymer, and EG-PEDOT:PSS-CNT additionally doped with EG. Analysis of the fracture morphology and rheological properties of the prepared nanocomposites revealed that all modified CNTs were well-dispersed in the PS matrix. The electrical conductivity of the PS/PEDOT:PSS-CNT nanocomposite was higher than that of the PS/PDA-CNT counterpart because the conductive polymer PEDOT:PSS helped form the electrical network. Furthermore, the secondary doping of PEDOT:PSS with EG resulted in a higher PEDOT-to-PSS ratio, thereby increasing the electrical conductivity. The electrical percolation thresholds of the nanocomposites with PDA-CNTs, PEDOT:PSS-CNTs, and EG-PEDOT:PSS-CNTs were 0.58, 0.32, and 0.36 wt%, respectively. The electrical conductivity of the nanocomposite prepared with an EG-PEDOT:PSS-CNT content of 4 wt% was as high as 10^0^ S m^−1^.

## Figures and Tables

**Table 1:** Sheet resistances of spin-coated PEDOT:PSS films.

Film Type	EG Content (wt%)	Coating Speed (rpm)	Sheet Resistance (Ω sq^−1^)
PEDOT:PSS	0	600	10,845
		2000	18,588
EG-PEDOT:PSS	2	600	360
		2000	1188
EG-PEDOT:PSS	5	600	240
		2000	801

## Data Availability

Not applicable.
